# Guanine nucleotide exchange factor DOCK11-binding peptide fused with a single chain antibody inhibits hepatitis B virus infection and replication

**DOI:** 10.1016/j.jbc.2022.102097

**Published:** 2022-06-02

**Authors:** Mayuko Ide, Noriko Tabata, Yuko Yonemura, Takayoshi Shirasaki, Kazuhisa Murai, Ying Wang, Atsuya Ishida, Hikari Okada, Masao Honda, Shuichi Kaneko, Nobuhide Doi, Satoru Ito, Hiroshi Yanagawa

**Affiliations:** 1 Research Department, Purotech Bio Inc, Yokohama, Kanagawa, Japan; 2 Department of Clinical Laboratory Medicine, Kanazawa University Graduate School of Health Medicine, Kanazawa, Ishikawa, Japan; 3 Department of Gastroenterology, Kanazawa University Graduate School of Medicine, Kanazawa, Ishikawa, Japan; 4 Department of Biosciences and Informatics, Keio University, Yokohama, Kanagawa, Japan

**Keywords:** hepatitis B virus, DOCK11, drug delivery, anti-HBV peptide drug, IVV method, peptide drug delivery, fusion protein, peptide transport, antibody engineering, ASGR, asialoglycoprotein receptor, cccDNA, covalently closed circular DNA, cDNA, complementary DNA, DHR, DOCK homology region, HBV, hepatitis B virus, HRP, horseradish peroxidase, IVV, *in vitro* virus, NLS, nuclear localization signal, NTCP, sodium-taurocholate cotransporting polypeptide, rcDNA, relaxed circular DNA, RIPA, radioimmunoprecipitation assay, TBS, Tris-buffered saline, TBST, TBS with Tween-20

## Abstract

Hepatitis B virus (HBV) infection is a major global health problem with no established cure. Dedicator of cytokinesis 11 (DOCK11), known as a guanine nucleotide exchange factor (GEF) for Cdc42, is reported to be essential for the maintenance of HBV. However, potential therapeutic strategies targeting DOCK11 have not yet been explored. We have previously developed an *in vitro* virus method as a more efficient tool for the analysis of proteomics and evolutionary protein engineering. In this study, using the *in vitro* virus method, we screened and identified a novel antiasialoglycoprotein receptor (ASGR) antibody, ASGR3-10M, and a DOCK11-binding peptide, DCS8-42A, for potential use in HBV infection. We further constructed a fusion protein (10M-D42AN) consisting of ASGR3-10M, DCS8-42A, a fusogenic peptide, and a nuclear localization signal to deliver the peptide inside hepatocytes. We show using immunofluorescence staining that 10M-D42AN was endocytosed into early endosomes and released into the cytoplasm and nucleus. Since DCS8-42A shares homology with activated cdc42-associated kinase 1 (Ack1), which promotes EGFR endocytosis required for HBV infection, we also found that 10M-D42AN inhibited endocytosis of EGFR and Ack1. Furthermore, we show 10M-D42AN suppressed the function of DOCK11 in the host DNA repair system required for covalently closed circular DNA synthesis and suppressed HBV proliferation in mice. In conclusion, this study realizes a novel hepatocyte-specific drug delivery system using an anti-ASGR antibody, a fusogenic peptide, and DOCK11-binding peptide to provide a novel treatment for HBV.

Hepatitis B virus (HBV) is a global public health problem, with more than 250 million people chronically infected worldwide. Chronic HBV infection is a leading cause of liver disease, including liver cirrhosis, hepatocellular carcinoma, and liver failure, and thus, HBV infection causes over 700,000 deaths annually (1, 2). HBV causes hepatocellular carcinoma, and chronic infection can lead to serious liver disease, such as liver fibrosis and cirrhosis (3, 4, 5). Although vaccines can prevent infection, chronic infection cannot be completely cured by vaccines or any other treatment. Therefore, it is essential to clarify the pathway of HBV infection and develop a new effective therapy for elimination of HBV.

The HBV replication system has been partially revealed. The virus attaches to the hepatocyte surface *via* several host receptors, such as sodium-taurocholate cotransporting polypeptide (NTCP) (6), and the viral nucleocapsid is imported inside the cell by endocytosis. The virion contains partially double-stranded relaxed circular DNA (rcDNA). During infection in the nucleus, rcDNA is converted to covalently closed circular DNA (cccDNA) *via* host DNA repair machinery (7, 8, 9). cccDNA is a transcript template for viral RNA, and its existence causes persistent infection (10). Translated viral proteins act as infectious viral particles or are recycled back to the nucleus.

DOCK11 has been identified as a host factor that is necessary for intracellular maintenance of HBV (11). DOCK family members function as guanine nucleotide exchange factors (GEFs), and DOCK11 is specific for Cdc42 (12). Cdc42 is a member of the Rho GTPase subfamily and regulates multiple cellular processes, including cell polarity and membrane trafficking, by regulating the actin cytoskeleton (13, 14). Although Cdc42 stimulates diverse cellular activities and is involved in the activation of HIV-1 and other viruses (15), the role of the interaction between DOCK11 and Cdc42 in HBV infection has not been investigated.

In this study, we used the *in vitro* virus (IVV) method (16, 17, 18) to identify novel DOCK11-binding peptides and antibodies against asialoglycoprotein receptor (ASGR). We identified the DOCK11-binding peptide DCS8-42A with anti-HBV activity, and the anti-ASGR antibody ASGR3-10M had a strong affinity for ASGR, which can be used for hepatocyte-specific uptake. Because glycoproteins with sialic acid, which are abundant in the blood, deteriorate into asialoglycoproteins and are degraded in the liver, the liver has ASGR (19, 20). The anti-ASGR antibody is expected to bind to receptors, followed by internalization into hepatocytes. Consequently, we fused DCS8-42A with ASGR3-10M to construct a fusion protein named 10M-D42AN that delivers the peptide into hepatocytes. We confirmed that DCS8-42A has homology to activated cdc42-associated kinase 1 (Ack1) and that 10M-D42AN inhibits the interaction between DOCK11, Cdc42, and Ack1 and the activation of actin polymerization and epidermal growth factor receptor (EGFR) endocytosis. Furthermore, 10M-D42AN suppressed HBV infection in mice.

## Results

### Construction of anti-ASGR antibody-fused DOCK11-binding peptide obtained by *in vitro* selection

DOCK11 is a member of the DOCK-D family, which interacts with Cdc42 (21). The DOCK-D family consists of DOCK9, DOCK10, and DOCK11. DOCK-D family proteins contain two regions whose amino acid sequences are well conserved among family members, DOCK homology region (DHR) 1 and DHR2, which are known to activate specific Rho family G proteins *via* DHR2 (22). Furthermore, Yang *et al.* (23) reported the crystal structure of the Cdc42–DOCK9 complex and revealed that DOCK9-interacting residues of Cdc42 are located in the N-terminal DHR2 domain. Since DOCK11 has high homology to DOCK9, it is possible that the DOCK11-interacting residues of Cdc42 are similar to those of DOCK9.

**Figure 1:**
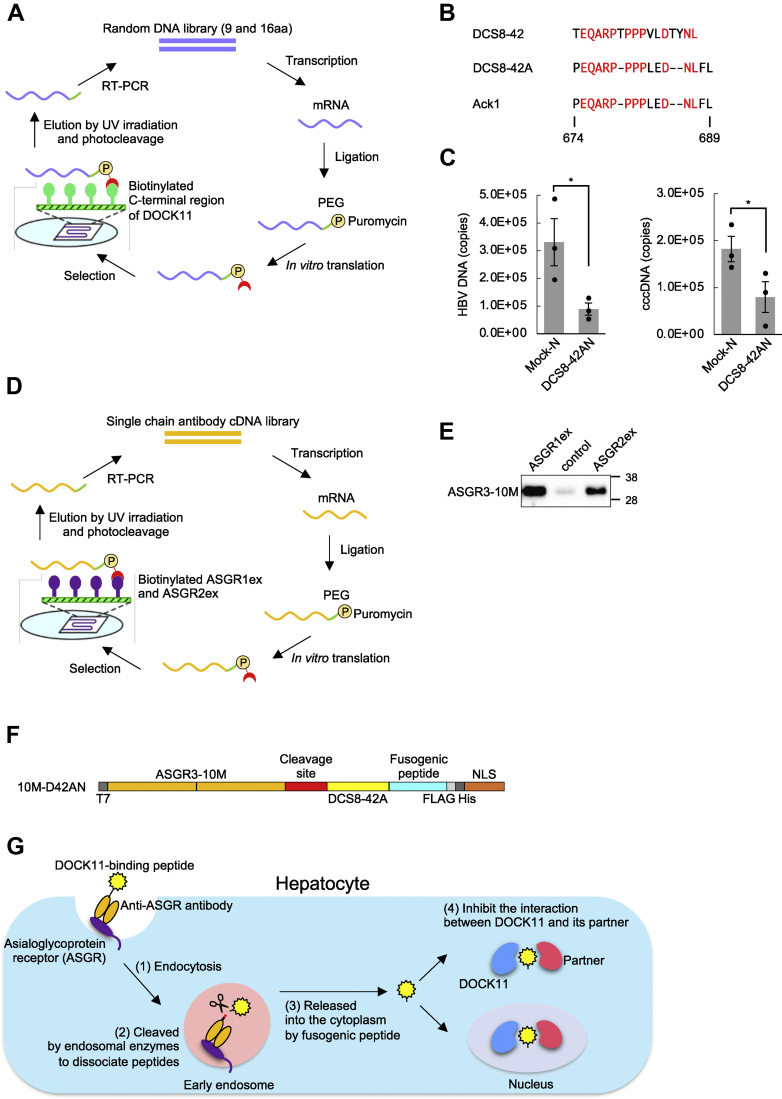
**Selection and mechanism of action of DOCK11-binding peptide fused with a single chain antibody.***A*, schematic representation of mRNA display for DOCK11-binding peptide. *Step 1*: A random cDNA library of 16 residues was transcribed and ligated with a PEG-puromycin spacer. *Step 2*: The resulting mRNA template was translated *in vitro* to form a library of protein-mRNA conjugates. *Step 3*: The library was injected into a microfluidic chip on which biotinylated DOCK11 was immobilized, and unbound molecules were washed away. *Step 4*: The bound molecules were eluted with 365 nm UV light, and their mRNA portion was amplified *via* RT-PCR. The resulting DNA was size-fractionated, and larger DNA fragments were used for the next round of selection. *Step 5*: After eight selection rounds, the enriched DNA was analyzed *via* cloning and sequencing. *B*, the amino acid sequence of the peptides identified as DOCK11-binding peptides by mRNA display and of Ack1 (674–689 aa). *C*, HepG2-NTCP-C4-Halo-DOCK11 cells were infected with HBV, induced to express DOCK11 with doxycycline and transfected with nuclear localization sequence (NLS)-tagged mock (Mock-N) or DCS8-42A (DCS8-42AN). HBV DNA and cccDNA were measured using qPCR. Data are presented as the mean ± SE pooled from three independent experiments. ∗*p* < 0.05. *D*, schematic representation of mRNA display for anti-ASGR antibody. *Step 1*: A cDNA library of single chain antibodies was transcribed and ligated with a PEG-puromycin spacer. *Step 2*: The resulting mRNA template was translated *in vitro* to form a library of protein-mRNA conjugates. *Step 3*: The library was injected into a microfluidic chip on which the biotinylated extracellular domains of ASGR1 and ASGR2 were immobilized, and unbound molecules were washed away. *Step 4*: The bound molecules were eluted with 365 nm UV light, and their mRNA portion was amplified *via* RT-PCR. The resulting DNA was size-fractionated, and larger DNA fragments were used for the next round of selection. *Step 5*: After three selection rounds, the enriched DNA was analyzed *via* cloning and sequencing. *E*, pull-down assay of ASGR3-10M with biotinylated ASGR1 or ASGR2. HEK293T cells were transfected with a construct encoding Igk-ASGR3-10M, and after 6 days, whole-cell lysates were prepared and used for pull-down assays with ASGR1ex- or ASGR2ex-immobilized or nonimmobilized beads. Each fraction was separated by gel electrophoresis using 4% to 12% Bis–Tris gels, followed by Western blot analysis using an antibody against the FLAG tag. The numbers on the right of the panels indicate the size markers of protein (kDa). *F*, design of 10M-D42AN, a fusion between DOCK11-binding peptide and anti-ASGR antibody. A cleavage site for an endosomatic enzyme, fusogenic peptide for release from early endosomes and NLS are also present in the fusion protein. *G*, mechanism of action of 10M-D42AN in hepatocytes. (1) 10M-D42AN is endocytosed into early endosomes *via* an anti-ASGR antibody. (2) 10M-D42AN is cleaved at the cleavage site by the enzyme Furin in endosomes, followed by dissociation of the peptide. (3) The peptide DCS8-42A is released into the cytoplasm *via* the fusogenic peptide. (4) DCS8-42A inhibits the interaction between DOCK11 and its partner proteins in the cytoplasm and nucleus (which it can enter *via* the NLS). ASGR, asialoglycoprotein receptor; cccDNA, covalently closed circular DNA; cDNA, complementary DNA; HBV, hepatitis B virus; qPCR, quantitative PCR; SE, standard error.

First, we performed IVV selection to obtain DOCK11-binding peptides. A schematic representation of the method is shown in Figure 1*A*. The DHR2 domain of DOCK11, which is required to interact with Cdc42, was used as bait. After four rounds of selection, we obtained a peptide, DCS8-42, that is homologous to Ack1 (674–689 aa; Fig. 1*B*) and then synthesized DCS8-42A, which is a part of the Ack1 sequence. We examined whether DCS8-42A has an anti-HBV effect on HepG2-NTCP-C4-Halo-DOCK11 cell lines, in which DOCK11 expression is induced by treatment with doxycycline (submitted to Cell. Mol. Gastroenterol. Hepatol.). HepG2-NTCP-C4-Halo-DOCK11 cells were infected with HBV, induced to express DOCK11, and then transfected with nuclear localization signal (NLS)–tagged DCS8-42A (DCS8-42AN). We then observed that DCS8-42AN mostly suppressed intracellular HBV DNA and cccDNA levels (Fig. 1*C*).

Next, IVV selection of an anti-ASGR antibody was performed using a single chain antibody (scFv) complementary DNA (cDNA) library (Fig. 1*D*). Biotinylated ASGR1ex and biotinylated ASGR2ex were prepared by adding biotin to the extracellular domains of ASGR1 and ASGR2, respectively, since the receptors ASGR1 and ASGR2 are present in human hepatocytes (24) and form heterooligomers. After three rounds of selection, we obtained scFv, named ASGR3-10M, which interacts with ASGR1 and ASGR2 (Fig. 1*E*).

We then constructed fusion protein 10M-D42AN consisting of scFv ASGR3-10M, a Furin cleavage sequence, DOCK11-binding peptide DCS8-42A, fusogenic peptide S28, and an NLS (Fig. 1*F*). Fusogenic peptide S28 is derived from the human protein syncytin-1, which is involved in cell membrane fusion during placental development. The cytosolic delivery of therapeutic proteins by cell-penetrating peptides, such as a HIV-derived TAT peptide, is known to be facilitated by fusogenic peptides (25). The previously discovered syncytin-1-derived peptide S19 needed to be adjacent to TAT (26), but when the C-terminal side of S19 was extended to S28, it showed activity even when separated from TAT (unpublished results). Here, we tested the combination of S28 with an anti-ASGR antibody to enhance the endosomal escape efficiency of the DOCK11-binding peptide incorporated by ASGR-dependent endocytosis. A schematic representation of the mechanism of action is shown in Figure 1*G*. The construct is expected to be endocytosed into early endosomes *via* the anti-ASGR antibody and then cleaved by the enzyme Furin in the endosome, followed by dissociation of the peptide. The peptide may be released into the cytoplasm *via* the fusogenic peptide and inhibit the interaction between DOCK11 and its partner proteins in the cytoplasm and nucleus.

### DCS8-42A exhibits anti-HBV activity in PXB cells

**Figure 2:**
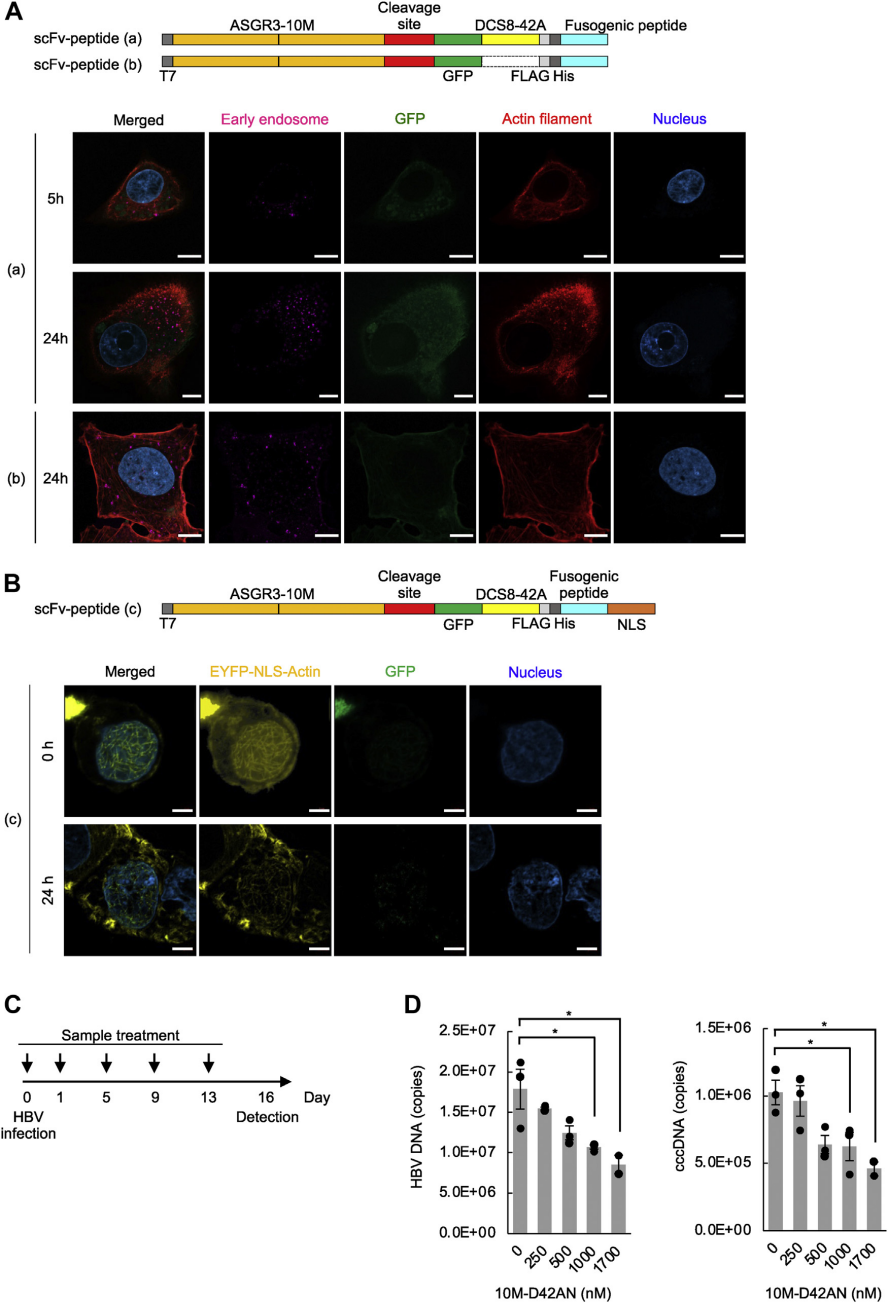
**10M-D42AN inhibited HBV infection in primary human hepatocytes.***A*, HepG2 cells were treated with scFv-peptide (a, b) containing or not containing DCS8-42A. After 5 to 24 h, immunofluorescence staining with CellLight Early Endosomes-RFP (*pink*) and rhodamine phalloidin (*red*) was performed. The samples were observed using confocal microscopy. The scale bars represent 10 μm. *B*, HepG2 cells transfected with EYFP-NLS-Actin were treated with scFv-peptide (c), which is GFP-tagged 10M-D42AN. After 24 h, the cells were observed using confocal microscopy. The scale bars represent 5 μm. *C*, schedule of HBV infection and the treatment of PXB cells with 10M-D42AN. *D*, PXB cells were treated with 10M-D42AN. HBV DNA and cccDNA were measured using qPCR. Data are presented as the mean ± SE pooled from three independent experiments. ∗*p* < 0.05. cccDNA, covalently closed circular DNA; HBV, hepatitis B virus; NLS, nuclear localization signal; qPCR, quantitative PCR; SE, standard error.

We examined whether 10M-D42AN would work as expected. HepG2 cells were treated with scFv-peptide (a, b) consisting of ASGR3-10M, the cleavage site, GFP, DCS8-42A, and fusogenic peptide S28 and then observed under a microscope (Fig. 2*A*). scFv-peptide (a, b) was localized in the cytoplasm in a time-dependent manner, and actin filaments were fragmented only if the construct contained DCS8-42A.

The function of the NLS was also examined. HepG2 cells were transfected with EYFP-NLS-actin and then treated with or without GFP-tagged 10M-D42AN (scFv-peptide (c)). In the nucleus, GFP-tagged 10M-D42AN localized near fragmented actin filaments (Fig. 2*B*). Cells transfected with NLS-tagged EYFP-actin showed fragmented and thin filaments after scFv-peptide (c) treatment, although the filaments were stable and long without treatment.

In the same way, the function of fusogenic peptide S28 was examined. HepG2 cells were treated with scFv-peptide (d) consisting of ASGR3-10M, the cleavage site, GFP, and DCS8-42A (Fig. S1). Early endosomes became huge, and scFv-peptide (d) was observed *via* the GFP tag at the same position. scFv-peptide (d) was rarely observed in the cytoplasm, and there was no fragmentation of actin filaments. scFv-peptide (d) without fusogenic peptide was not released into the cytoplasm but accumulated in the endosomes.

The function of the cleavage site was investigated using scFv-peptide (a). scFv-peptide (a) was mixed with Furin protease at 37 °C for 30 min, and the cleaved N-terminal and C-terminal regions were detected (Fig. 2, *A* and *B*). In addition, when scFv-peptide (a) was reacted with human blood at 37 °C for 0 to 6 h, the truncated C-terminal region increased in a time-dependent manner. After 6 h, approximately 30% of the truncated region was observed (Fig. S2*C*).

Finally, we examined whether 10M-D42AN inhibits HBV infection in PXB primary human hepatocytes. PXB cells were treated with 10M-D42AN, and HBV infection was assessed by measuring HBV DNA and cccDNA levels (Fig. 2*C*). 10M-D42AN was found to decrease HBV infection in a concentration-dependent manner (Fig. 2*D*). IC_50_ values of 10M-D42AN for inhibiting proliferation of HBV DNA and cccDNA were 1.5 μM and 1.4 μM, respectively. These results confirmed that the scFv-fused peptide works in hepatocytes, as expected.

### DCS8-42A inhibits the GEF activity of DOCK11 and the interaction between DOCK11 and Ack1

Subsequently, we investigated the mechanism of action of 10M-D42AN. DOCK11 exhibits GEF activity for Cdc42 *via* its DHR2 domain (12). Unlike other GEF proteins, DOCK11 interacts with activated GTP-bound Cdc42 and provides positive feedback for Cdc42 activation. The nonreceptor tyrosine kinase Ack1 was reported to specifically interact with GTP-bound Cdc42 and not other small G-proteins (27). Thus, we examined whether DCS8-42A inhibits the GEF activity of DOCK11 since Ack1 may compete with DOCK11 for the interaction between GTP-bound Cdc42 to suppress further activation of Cdc42.

**Figure 3:**
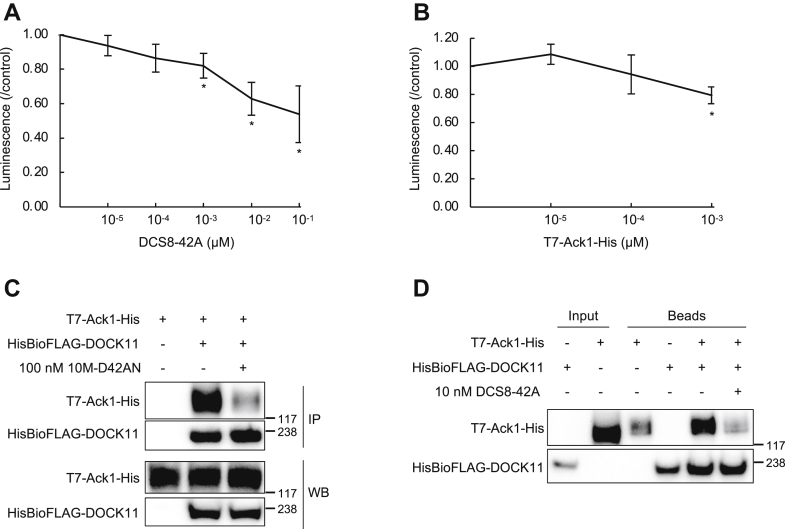
**DCS8-42AN inhibited the GEF activity of DOCK11 and its interaction with Ack1**. *A* and *B*, GEF assay with DCS8-42A (*A*) or Ack1 (*B*). GDP-bound Cdc42 was added to the DHR2 domain of DOCK11 immobilized on a streptavidin plate in the presence of various concentrations of DCS8-42A (*A*) or T7-Ack1-His (*B*) purified from the lysate. The plate was finally treated with Mant-GTP. ∗*p* < 0.05. *C*, coimmunoprecipitation assay using T7-Ack1-His and HisBioFLAG-DOCK11 coexpressed in HEK293T cells treated with 10M-D42AN. The lysates were immunoprecipitated and immunoblotted using anti-Ack1 or anti-FLAG antibody. *D*, pull-down assays in the presence of 100 nM DCS8-42A using T7-Ack1-His and HisBioFLAG-DOCK11. HEK293T cells were transfected with a construct encoding T7-Ack1-His, and whole-cell lysates were prepared and used for pull-down assays with HisBioFLAG-DOCK11-immobilized beads. Each fraction was separated in a 3% to 8% SDS-PAGE and then analyzed by Western blotting with an antibody against Ack1 or FLAG-tag. DHR, DOCK homology region.

We performed a GEF assay using amino acid residues 1516 to 2073 of DOCK11, including its DHR2 domain. The DHR2 domain of DOCK11 was immobilized on streptavidin plates and treated with GDP-bound Cdc42 in the presence of 0 to 10 pmol of DCS8-42A. The plate was finally treated with Mant-GTP, whose fluorescence intensity is increased by binding to proteins such as Cdc42. We found that DCS8-42A inhibited the GEF assay of DOCK11 in a concentration-dependent manner (Fig. 3*A*). Ack1 also suppressed GEF activity *in vitro* (Fig. 3*B*).

Next, we examined whether overexpressed Ack1 and DOCK11 interact with each other in cells and whether 10M-D42AN inhibits the interaction. We performed a coimmunoprecipitation assay of T7-tagged Ack1 and FLAG-tagged DOCK11 in total lysates of cells treated with 10M-D42AN. The results showed that Ack1 bound to DOCK11 in cells and that the interaction was inhibited by 10M-D42AN (Fig. 3*C*). Additionally, we examined whether DCS8-42A inhibits the interaction between Ack1 and DOCK11 *in vitro*. We performed a binding assay of T7-tagged Ack1 using beads bearing DOCK11 in the presence of DCS8-42A. Western blot analysis of each bead fraction showed that Ack1 bound to DOCK11 and that this interaction was inhibited by DCS8-42A (Fig. 3*D*).

### 10M-D42AN inhibits actin polymerization

**Figure 4:**
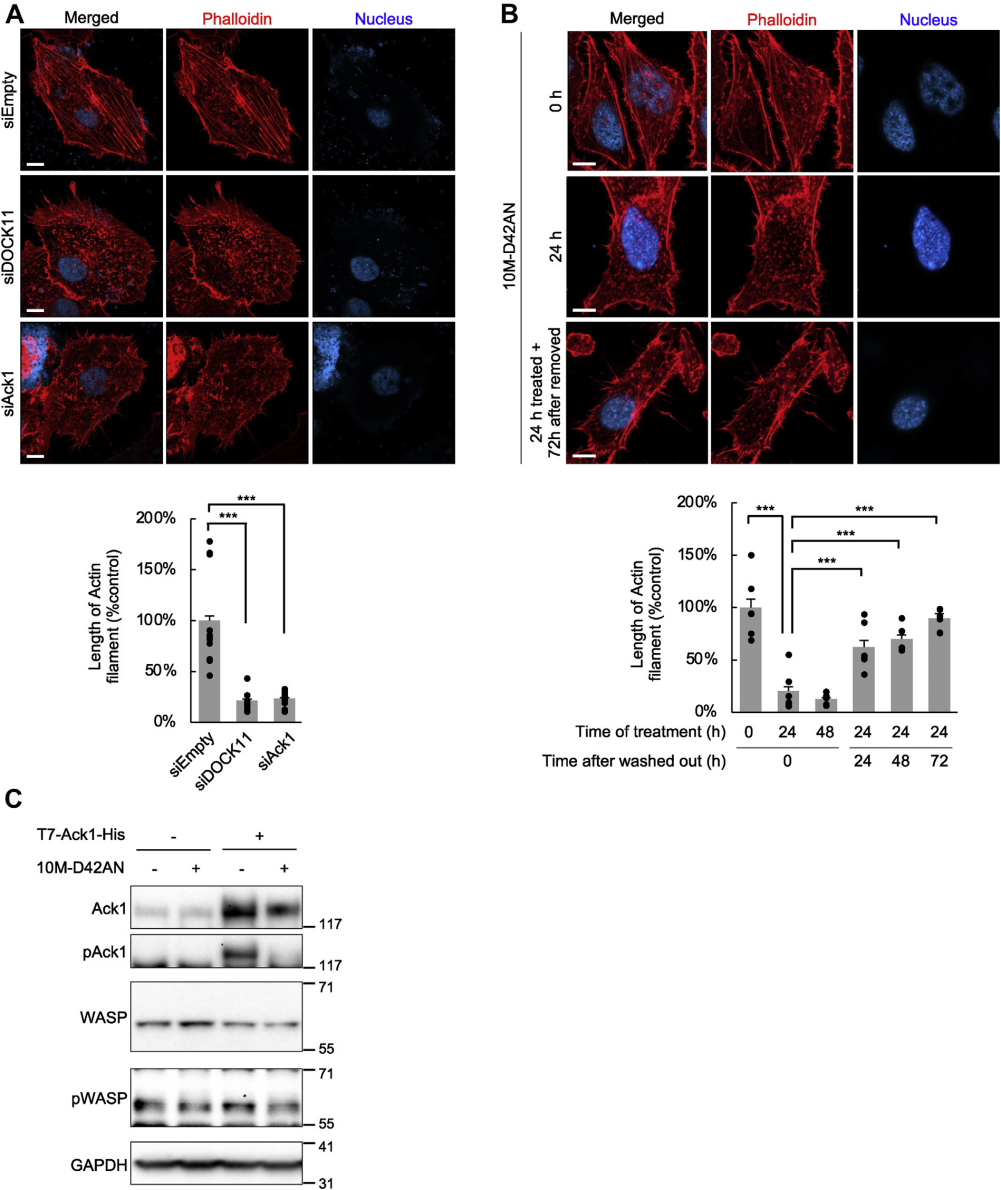
**10M-D42AN inhibited actin polymerization.***A*, phalloidin staining of actin filaments in HepG2 cells treated with siRNA targeting DOCK11 or Ack1. The samples were observed using confocal microscopy. The scale bars represent 10 μm. *B*, phalloidin staining for actin filaments in HepG2 cells treated with 10M-D42AN for 24 to 48 h and then incubated in the usual medium for another 24 to 72 h. The samples were observed using confocal microscopy, and the length of actin filaments was measured. Error bars represent SE. ∗∗∗*p* < 0.0005. The scale bars represent 10 μm. *C*, expression or phosphorylation level of Ack and WASP in HepG2 cells treated with 0 to 100 nM 10M-D42AN for 24 h. Whole-cell lysates were separated in a 3% to 8% SDS-PAGE and analyzed by Western blotting with antibodies against Ack1, pAck1, WASP, and pWASP. GAPDH is shown to verify equal loading.

The aforementioned experiments showed that 10M-D42AN has the ability to fragment actin cytoskeleton (Fig. 2, *A* and *B*). Activated Ack1 acts as a kinase for WASP and promotes actin polymerization (28). We performed knockdown experiments targeting DOCK11 or Ack1 to confirm the effect of these proteins on actin filaments. As shown in Fig. S3, *A* and *B*, siRNA-mediated knockdown of endogenous Ack1 did not affect the amount of *DOCK11* mRNA, although knockdown of endogenous DOCK11 increased the expression of Ack1. Cytoskeletal F-actin staining showed that the knockdown of DOCK11 or Ack1 increased the number of fragmented cytoplasmic actin filaments and filopodia (Fig. 4*A*).

We also examined the effect of 10M-D42AN on actin polymerization. Treatment with 10M-D42AN did not affect *DOCK11* mRNA (Fig. 3, *C* and *D*). HepG2 cells treated with 10M-D42AN for 24 h were washed and then incubated with their usual medium for up to 72 h. Cytoskeletal F-actin staining showed that treatment with 10M-D42AN fragmented actin filaments and that washout and additional incubation for 72 h completely recovered the shortened filaments (Fig. 4*B*). This result shows that 10M-D42AN affects the formation of actin filaments in a similar manner to knockdown of DOCK11 or Ack1, although the effect was plastic.

We further examined the phosphorylation of Ack1 and WASP in the presence of 10M-D42AN. HepG2 cells transfected with T7-Ack1-His were treated with 0 or 100 nM 10M-D42AN, and then, a Western blot analysis was performed. The results showed that Ack1 and WASP were phosphorylated with overexpression of Ack1 in the absence of 10M-D42AN treatment but were not activated in the presence of 10M-D42AN (Fig. 4*C*).

### 10M-D42AN inhibits endocytosis of EGFR

**Figure 5:**
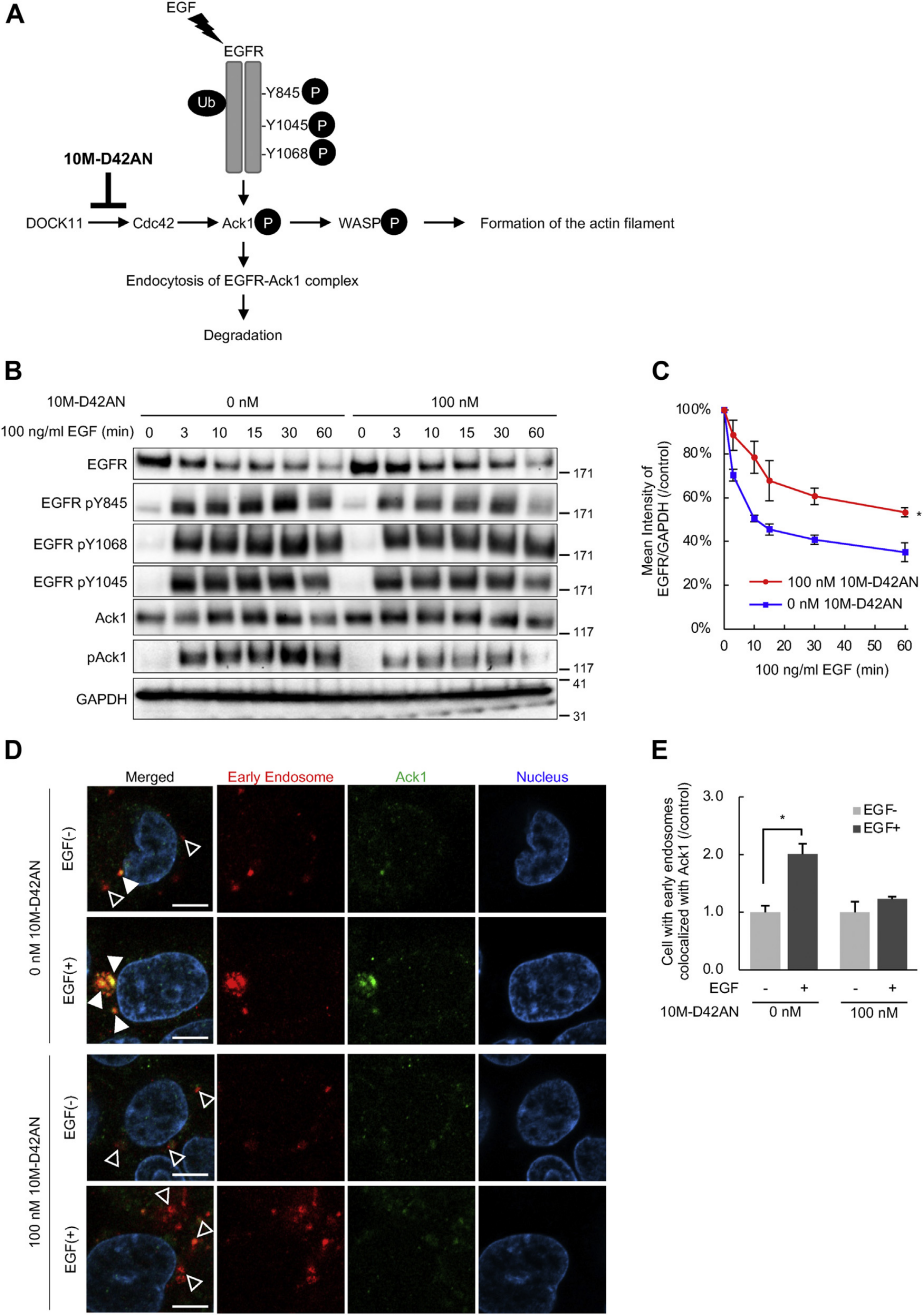
**10M-D42AN inhibited endocytosis of EGFR and Ack1.***A*, schematic representation of the mechanism of action of 10M-D42AN in EGFR endocytosis. Upon EGF stimulation, EGFR is phosphorylated and ubiquitinated and then interacts with Ack1 activated by Cdc42, which is activated by DOCK11. The EGFR–Ack1 complex is endocytosed into early endosomes and then degraded. On the other hand, activated Ack1 promotes the formation of actin filaments through the phosphorylation of WASP. Since 10M-D42AN inhibits Cdc42 activation, the activation of Ack1 and endocytosis of the EGFR–Ack1 complex are suppressed, although the phosphorylation of EGFR is not affected. *B*, expression or phosphorylation level of Ack and EGFR in Huh7 cells treated with 0 to 100 nM 10M-D42AN for 24 h and 100 ng/ml EGF for 0 to 60 min. Whole-cell lysates were separated in a 3% to 8% SDS-PAGE and analyzed by Western blotting with antibodies against Ack1, pAck1, EGFR, and EGFR (pY845, pY1045, pY1068). GAPDH is shown to verify equal loading. *C*, the expression level of EGFR shown in *B*. Data are presented as the mean ± SE pooled from three independent experiments. ∗*p* < 0.05. *D*, immunofluorescence staining of HepG2 cells using anti-Ack1 antibody (Alexa488, *green*) after treatment with 0 to 100 nM 10M-D42AN for 24 h and 100 ng/ml EGF for 60 min. Early endosome staining with CellLight Early Endosomes-RFP (*red*) is also shown. The samples were observed under a fluorescence microscope. Early endosomes colocalized with Ack1 are indicated by *white arrows*; otherwise, they are indicated by *white arrowheads*. The scale bars represent 10 μm. *E*, colocalization of Ack1 and early endosomes in the cells was scored for 150 to 250 cells. The ratio under the indicated conditions is shown. Error bars represent SE. ∗*p* < 0.05. SE, standard error.

It has been reported that endocytosis of EGFR is essential for HBV entry with NTCP (6). Ack1 is also reported to be involved in EGFR endocytosis (29, 30, 31). Ack1 activated by the interaction with Cdc42 interacts with EGFR at phosphorylated Tyr1045 upon EGF stimulation, and both are transported to early endosomes together. Therefore, we investigated whether 10M-D42AN inhibits EGFR endocytosis (Fig. 5*A*).

First, we examined whether 10M-D42AN inhibits the activation and degradation of EGFR. Huh7 cells were treated with 10M-D42AN for 24 h and with 100 ng/ml EGF for 0 to 60 min, followed by Western blotting. As previously reported, Ack1 was phosphorylated upon EGF stimulation, although this activation was inhibited by treatment with 10M-D42AN (Fig. 5*B*). The activation of EGFR detected with phosphorylation of Tyr845, Tyr1045, and Tyr1068 did not change in the presence of 10M-D42AN. However, the EGF-stimulated decrease in EGFR expression was inhibited by 10M-D42AN treatment (Fig. 5*C*).

Next, we examined the effect of 10M-D42AN on the localization of Ack1 and EGFR in early endosomes. HepG2 cells expressing CellLight Early Endosomes-RFP were treated with 0 to 100 nM 10M-D42AN for 24 h and 100 ng/ml EGF for 1 h. Immunofluorescence staining followed by confocal fluorescence microscopy showed that the localization of Ack1 in early endosomes was increased with EGF stimulation (Fig. 5, *D* and *E*). However, Ack1 was not localized in early endosomes in the cells treated with 10M-D42AN. These results indicate that 10M-D42AN inhibits Ack1 activation and subsequent endocytosis of the Ack1–EGFR complex to early endosomes.

### DOCK11 mediates ATR signaling in DNA repair and 10M-D42AN inhibits it

cccDNA of HBV is converted from rcDNA *via* the host DNA damage repair system, specifically, the ataxia telangiectasia and Rad3-related protein (ATR)-Chk1 signaling pathway not the ataxia telangiectasia mutated (ATM) pathway (8). ATR is recruited to DNA damage sites through nuclear actin assembly (32). It has been reported that the binding of Pleckstrin homology domain–containing proteins to PIP_3_ accumulated on DNA damage sites is followed by nuclear actin assembly and subsequent activation of the ATR signaling pathway. DOCK11 contains the Pleckstrin homology domain, suggesting that DOCK11 may be essential for DNA repair and thus cccDNA synthesis by activating Cdc42 at DNA damage sites and promoting actin formation.

**Figure 6:**
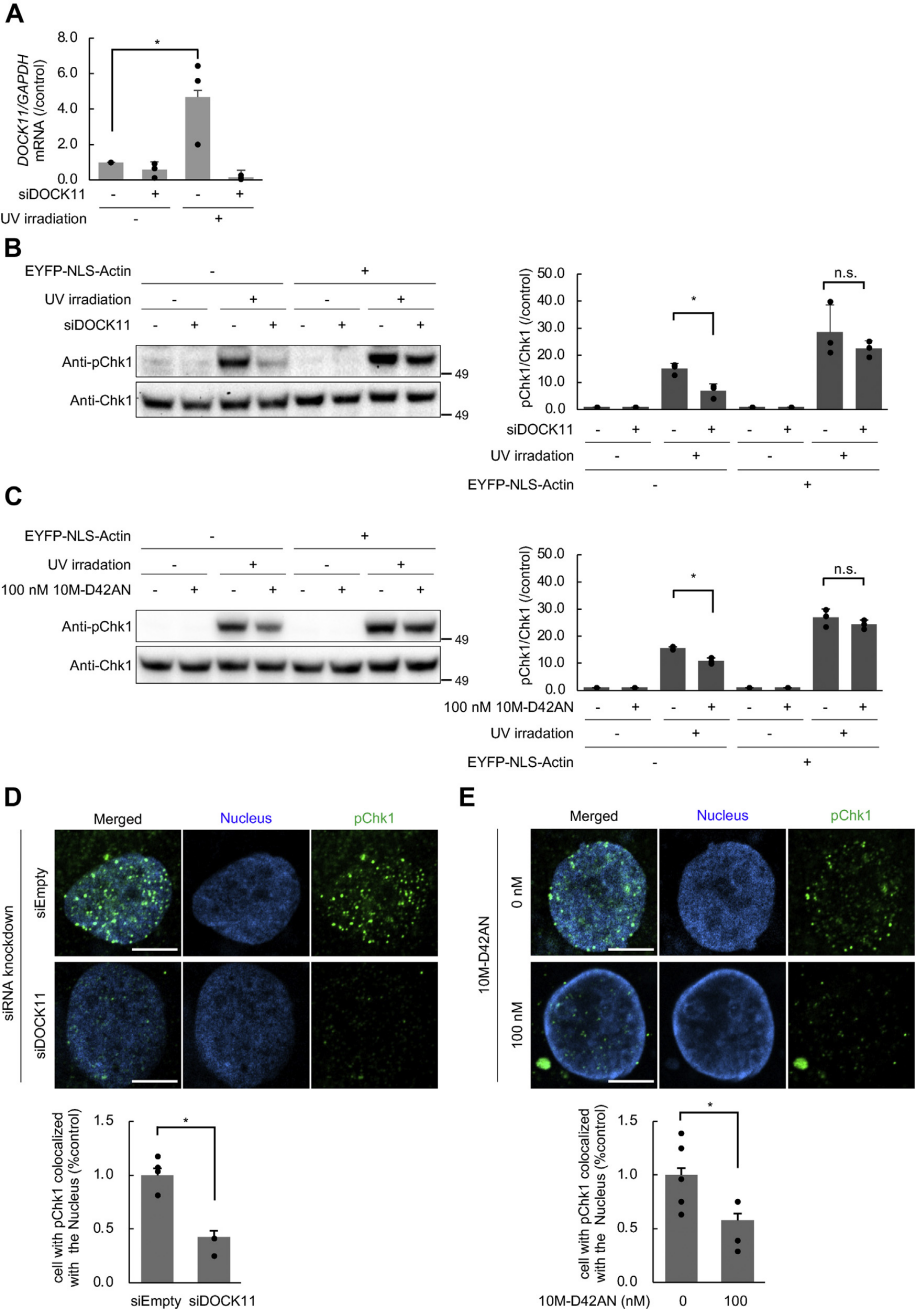
**10M-D42AN inhibited ATR signaling pathway mediated by DOCK11.***A*, the amount of *DOCK11* mRNA in HepG2 cells treated with siRNA targeting DOCK11 and irradiated with UV light. Total RNA was extracted and then amplified *via* RT-PCR with specific primers for *GAPDH* or *DOCK11*. Data are presented as the mean ± SE pooled from three independent experiments. ∗*p* < 0.05; n.s.: not significant. *B* and *C*, HepG2 cells were transfected with siRNA targeting DOCK11 and EYFP-NLS-Actin (*B*), or HepG2 cells transfected with EYFP-NLS-Actin were treated with 100 nM 10M-D42AN for 24 h (*C*). these cells were then irradiated with UV light, and the whole-cell lysates were separated in a 4% to 12% SDS-PAGE and analyzed by Western blotting with antibodies against Chk1 and pChk1. The band intensity for pChk1 was quantified and normalized to that for Chk1. ∗*p* < 0.05; ∗∗*p* < 0.005; n.s.: not significant. *D* and *E*, immunofluorescence staining of HepG2 cells using anti-pChk1 antibody (Alexa488, *green*) after treatment with siRNA targeting DOCK11 (*D*) or 0 to 100 nM 10M-D42AN for 24 h (*E*). the samples were observed under a fluorescence microscope. The scale bars represent 10 μm. Localization of pChk1 in the nucleus was scored for over 100 cells. The ratio under the indicated conditions is shown. Error bars represent SE. ∗*p* < 0.05. NLS, nuclear localization signal; SE, standard error.

First, we examined whether DOCK11 is involved in the ATR signaling pathway. The transcription level of DOCK11 and the phosphorylation level of Chk1 were drastically increased upon UV irradiation in HepG2 cells (Fig. 6*A*). Knockdown of DOCK11 strongly suppressed the increase in the *DOCK11* mRNA level and Chk1 phosphorylation level upon UV irradiation, although expression of NLS-actin rescued the phosphorylation of Chk1 to the same level as that without DOCK11 knockdown (Fig. 6, *A* and *B*). Since phosphorylation of Chk1 at Ser345 is a signal of the activation of ATR signaling pathway (33, 34), these results indicate that DOCK11 is involved in the ATR-dependent DNA damage response through the accumulation of nuclear actin. Treatment with 10M-D42AN before UV irradiation suppressed Chk1 phosphorylation, although the level of *DOCK11* mRNA was not changed (Fig. 6*C*). Immunofluorescence staining using an anti-pChk1 antibody also showed that knockdown of DOCK11 or treatment with 10M-D42AN inhibited the phosphorylation of Chk1 in the nucleus after UV irradiation (Fig. 6, *D* and *E*). These results indicate that DOCK11 is involved in the activation of ATR signaling and that 10M-D42AN inhibits ATR signaling through the inhibition of DOCK11.

**Figure 7:**
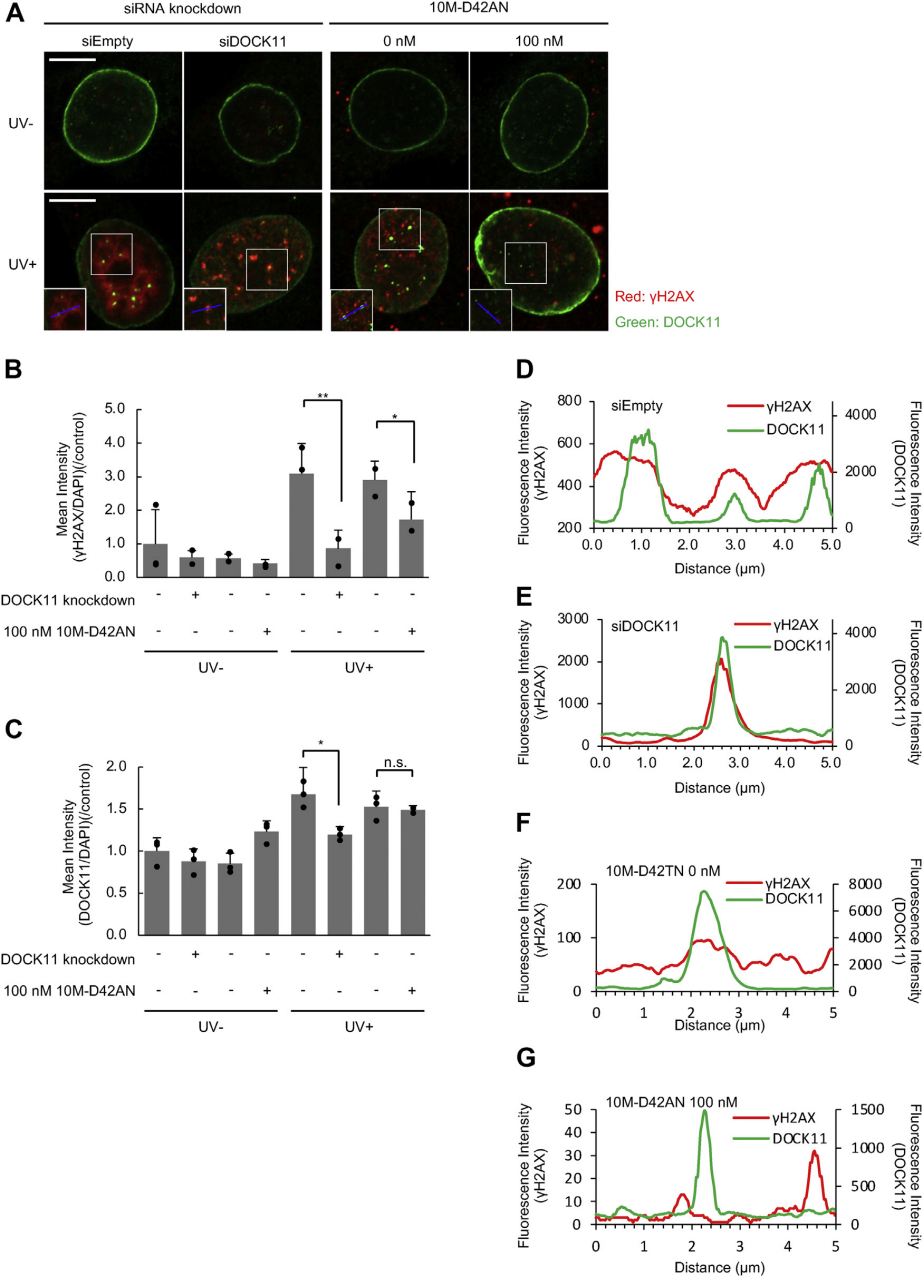
**PXB cells were treated with siRNA targeting DOCK11 or with 0 to 100 nM 10M-D42AN for 24 h and irradiated with UV light.***A*, immunofluorescence staining using anti-DOCK11 (Alexa488, *green*) or γH2AX (Alexa647, *red*) antibody. The samples were observed under a fluorescence microscope. The intensity profiles of DOCK11 and γH2AX shown in *D*–*G* are indicated by the *blue lines* in the inset image. The scale bars represent 10 μm. *B* and *C*, the fluorescence intensity of γH2AX (*B*) or DOCK11 (*C*) in the cells was quantified and normalized to that of DAPI. The ratio under the indicated conditions is shown. Error bars represent SE. ∗*p* < 0.05; ∗∗*p* < 0.005; n.s.: not significant. *D*–*G*, intensity profiles of γH2AX and DOCK11 along the straight *blue line* from the inset image in *A*. *Red* (γH2AX) and *green* (DOCK11) fluorescence intensities were measured for each pixel along a 5 μm *horizontal line* containing the *arrow tip* using the profile function in ZEN software. DAPI, 4′,6-diamidino-2-phenylindole; SE, standard error.

Next, we performed immunofluorescence staining to examine whether DOCK11 localizes to the DNA damage site. PXB cells were treated with 0 or 100 nM 10M-D42AN followed by immunostaining with an antibody against DOCK11. As a result, DOCK11 condensed in the nucleus upon UV irradiation, although it was normally localized at the nuclear lamina (Fig. 4, *A* and *B*). However, treatment with 10M-D42AN inhibited the condensation of DOCK11. We also examined colocalization of DOCK11 and γH2AX. PXB cells with knockdown of DOCK11 or treated with 10M-D42AN were immunostained with antibodies against DOCK11 and γH2AX. UV irradiation increased γH2AX in the nucleus, and this increase was suppressed by knockdown of DOCK11 or treatment with 10M-D42AN (Fig. 7, *A* and *B*), even though 10M-D42AN did not affect the expression level of DOCK11 (Fig. 7*C*). Interestingly, a decrease in the DOCK11 protein level promoted colocalization between the remaining DOCK11 and γH2AX (Fig. 7, *D* and *E*), while treatment with 10M-D42AN reduced the colocalization (Fig. 7, *F* and *G*). These results show that 10M-D42AN inhibits DOCK11 from directly localizing to DNA damage sites to activate the ATR signaling pathway.

### 10M-D42AN strongly inhibits HBV proliferation with entecavir

**Figure 8:**
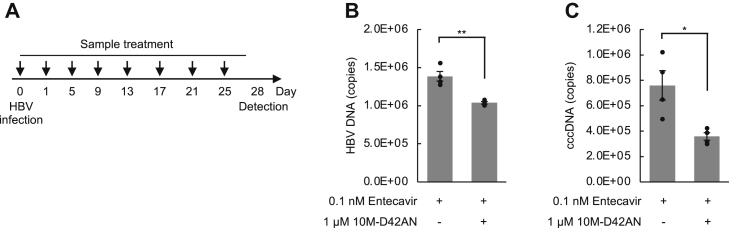
**10M-D42AN inhibited HBV proliferation with entecavir.***A*, schedule of HBV infection and treatment with 10M-D42AN and entecavir in PXB cells. *B* and *C*, HBV DNA (*B*) and cccDNA (*C*) in PXB cells treated with 10 nM entecavir in the absence or presence of 10 μM 10M-D42AN were detected by PCR. Error bars represent SE. ∗*p* < 0.05; ∗∗*p* < 0.005. cccDNA, covalently closed circular DNA; HBV, hepatitis B virus; SE, standard error.

We examined the anti-HBV effect of a combination of entecavir and 10M-D42AN in PXB cells. Entecavir is a nucleic acid analog that is currently used to treat HBV and is effective in suppressing the disease, although it does not cure it. PXB cells were infected with HBV, followed by treatment with entecavir in the absence or presence of 10M-D42AN for 28 days (Fig. 8*A*). The results showed that 10M-D42AN administered with entecavir reduced HBV DNA and cccDNA levels compared to entecavir alone (Fig. 8, *B* and *C*).

Further, we examined whether 10M-D42AN could inhibit HBV proliferation using PXB mice. PXB mice were transplanted with human hepatocytes and infected with HBV 56 days prior to the assay (Fig. S5*A*). 10M-D42AN was administered intraperitoneally every 2 to 3 days; livers were collected and assayed after 28 days. Sufficient amounts of human albumin were obtained from the chimeric mice in all assay periods, confirming that this assay system shows results in human liver cells. Alanine aminotransferase measurement showed that 10M-D42AN did not aggravate hepatitis compared to the control (Fig. S5*B*). HBV DNA and cccDNA levels in the liver also tended to decrease after 10M-D42AN administration (Fig. 5, *C* and *D*). Although modifications to the experimental design (*e.g.*, longer dosing periods, combination with entecavir) may improve the efficacy of 10M-D42AN, the results suggest that our drug delivery system is safe in mice.

## Discussion

HBV infection utilizes many host cell mechanisms, such as endocytosis of EGFR (6), the intracellular transport pathway to the nucleus (35, 36) and the DNA repair system (8, 9). Each mechanism is required for HBV attachment and entry, transport to the nucleus, and formation of cccDNA. DOCK11 is reported to be the GEF for Rho GTPase Cdc42 (12) and identified as an essential host factor that mediates HBV infection (11). Although Cdc42 also affect HBV proliferation and DOCK11-Cdc42 signaling pathway is indicated to be essential in the HBV infection (11), the role of DOCK11 activity with Cdc42 has not been clarified.

Yanagawa *et al.* (37) have demonstrated that the IVV method is very useful for the analysis and creation of various protein functions. For example, when proteins, peptides, and antigens are used as baits and screened from cDNA or random DNA libraries using the IVV method, protein complexes (17, 38, 39, 40, 41), signal sequences, physiologically active peptides (42, 43, 44, 45, 46), and antibodies with high affinity (18, 47) can be selected. Therefore, in this study, to investigate how DOCK11 mediates HBV infection, using the IVV method, we screened for peptides that inhibit the interaction between DOCK11 and Cdc42. As a result, we were able to obtain the peptide DCS8-42A, which showed homology to Ack1 (aa 674–689). Ack1 interacts with GTP-bound Cdc42 and promotes cell migration, actin polymerization, and EGFR endocytosis. We found that Ack1 regulates the activation of Cdc42 by interacting with DOCK11. It has been reported that DOCK11 binds to both GDP- and GTP-bound forms of Cdc42 in a positive feedback loop for Cdc42 activation (12). Although Ack1 interacts only with GTP-bound Cdc42 (48), in this study, Ack1 was found to bind to DOCK11 and inhibit its GEF activity. These results suggest that Ack1 has a regulatory role in preventing further Cdc42 activity. DCS8-42A inhibited the binding of Ack1 to DOCK11 and suppressed the GEF activity of DOCK11.

To determine the effect of DCS8-42A in hepatocytes, we generated a fusion protein, 10M-D42AN, with an anti-ASGR single chain antibody. The fusion was designed with the recognition sequence of an endosomal cleavage enzyme, a fusogenic peptide, and a nuclear translocation signal to allow the peptide to separate from the fusion once it enters the cell and function in the nucleus and cytoplasm. The results showed that 10M-D42AN inhibited endocytosis of Ack1 and EGFR and inhibited DOCK11 in the DNA repair system. Since HBV has been reported to utilize endocytosis of NTCP and EGFR when it enters hepatocytes (6), 10M-D42AN is suggested to inhibit HBV internalization by inhibiting endocytosis of Ack1 and EGFR. It has also been reported that HBV synthesizes cccDNA from rcDNA using ATR signaling pathway in the host DNA repair system (8), and it is known that the accumulation of actin filaments at sites of DNA damage is required for ATR assembly (32). Our experiments revealed that DOCK11 is involved in actin polymerization and colocalized with γH2AX, the marker for DNA damage site, to contribute to the activation of ATR signaling pathway. These results suggest that DOCK11 accumulates at DNA damage sites in response to DNA damage signals and promotes actin polymerization, thereby activating the ATR signaling pathway. In HBV-infected cells, DOCK11 may activate the ATR signaling pathway in this manner, thereby activating cccDNA formation. Since 10M-D42AN inhibited the colocalization of DOCK11 with γH2AX and actin polymerization in nuclear, it has a potential to inhibit HBV replication. Further investigation is needed to determine how DOCK11 is involved in the DNA repair system and whether DOCK11 is essential for the synthesis of cccDNA.

Rho GEFs and Rho GTPases have been reported to regulate the DNA damage response and replication in the nucleus; thus, the DOCK180 family is likely to be involved in this regulation. DOCK2 regulates the activity of the DNA damage response pathway by acting as a GEF for Rac1 and Rac2 (49). DOCK7 is phosphorylated by ATR and promotes the accumulation of RPA1 at replication forks through activation of the Rac1/Cdc42-PAK1 pathway (50). We have shown that DOCK11 regulates ATR activity through its GEF activity for Cdc42, thereby regulating the DNA damage response pathway. The DOCK180 family is structurally divided into four subfamilies, and DOCK2, DOCK7, and DOCK11 belong to different subfamilies (21, 22). DNA regulation through GEF activity may be a common function of DOCK180 family members.

Cell-based assays showed fragmentation of intracellular actin filaments by 10M-D42AN, which was restored by removal of the drug and had no effect on cell viability or cell adhesion; survival of PXB mice treated with 10M-D42AN was also unaffected, suggesting that long-term fragmentation of actin filaments is avoided by metabolism. The possibility of affecting other biological functions will need to be investigated in detail in the future, as well as the possibility of changing the target or adding a functional sequence based on the knowledge obtained here. Furthermore, a combination treatment with 10M-D42AN and entecavir in PXB cells inhibited HBV proliferation more than the treatment with entecavir alone. Therefore, 10M-D42AN is expected to be the basis for curative treatment of HBV, which is not currently available.

In conclusion, we constructed a hepatocyte-specific drug delivery system using an anti-ASGR antibody. Although DCS8-42 is an optimized sequence for binding to DOCK11 because we have performed IVV selection by introducing random mutations to obtain the DOCK11-binding peptide, we assessed the function of DCS8-42A, a partial sequence of Ack1, focusing on the function of DOCK11 and Ack1 in this study. Based on the findings of this study, optimization of the peptide sequence (*e.g.*, use of other sequences derived from Ack1, further modification by IVV selection from D42A) may more effectively inhibit the DOCK11–Ack1 interaction. The possibility that D42A interacts with other proteins related to DOCK11 and Ack1 should also be investigated in the future. We intend to change the composition of 10M-D42AN (for example, by changing the current mouse antibody to a human antibody that can be administered to humans) to obtain more efficient anti-HBV activity. Furthermore, this technology can be applied to functional peptides that bind to other proteins, and further developments in HBV research may lead to higher therapeutic efficacy.

## Experimental procedures

### Cell culture

HEK293T cells were maintained in Dulbecco's modified Eagle's medium (DMEM) (Nacalai Tesque) supplemented with 10% fetal bovine serum (Sigma) and 1% penicillin and streptomycin in a 5% CO_2_ incubator at 37 °C. HepG2 and Huh7 cells were cultured in DMEM supplemented with 10% fetal bovine serum, 2 mM L-glutamine (Nacalai Tesque), 1 mM sodium pyruvate (Nacalai Tesque), and 1% penicillin and streptomycin. Stable HepG2-NTCP-C4-Halo-DOCK11 cells in which DOCK11 was overexpressed *via* doxycycline administration were cultured in DMEM supplemented with 10% fetal bovine serum, 1% L-glutamine (Thermo Fisher Scientific), 100 U/ml penicillin, and 100 μg/ml streptomycin in a 5% CO_2_ incubator at 37 °C. PXB cells were purchased from PhoenixBio and cultured in DMEM supplemented with 10% fetal bovine serum, 20 mM Hepes, 15 μg/ml L-proline, 0.25 μg/ml insulin, 50 nM dexamethasone, 44 mM NaHCO_3_, 5 ng/ml EGF, 0.1 mM ascorbic acid 2-phosphate, 2% dimethyl sulfoxide, 100 U/ml penicillin, and 100 μg/ml streptomycin.

### HBV infection

HBV infection of PXB cells (primary human hepatocytes) purchased from PhoenixBio Co was performed as previously described (11). Briefly, PXB cells were cultured without passage. We prepared the culture medium with HBV particles with 5 GEq/cell, 10M-D42AN, and entecavir (Tokyo Chemical Industry) at the described final concentrations. After incubation for 20 to 28 h, the medium was removed, and the wells were washed once with PBS (Thermo). Then, the cells were cultured with medium containing 2% dimethyl sulfoxide and each drug. This medium exchange was performed 1, 5, 9, 13, 17, 21, and 25 days after the day of infection. On Day 16 or 28, the cells were collected to estimate the infection. HBV DNA and cccDNA were quantified using quantitative PCR.

### Preparation of plasmids

All primer sequences used in this study are listed in Table S1. A DNA clone of DOCK11 was purchased from Promega. His-tag, FLAG-tag, and Bio-tag sequences were amplified from pET101/D-TOPO (Invitrogen), pCMV6-LIPG (OriGene), and BioEase vector (Invitrogen), respectively, followed by cloning into pcDNA3.3 (Invitrogen) using an In-Fusion Cloning Kit (Clontech) to generate empty vectors named BioFLAGHis-pcDNA or HisBioFLAG-pcDNA. The full-length or 1516 to 2073 amino acid sequence of the DOCK11 gene was amplified *via* PCR and cloned into the BioFLAGHis-pcDNA vector to form DOCK11-BioFLAGHis-pcDNA or DOCK11-DHR2-BioFLAGHis-pcDNA. The extracellular domains of ASGR1 (62–291 aa) or ASGR2 (80–311 aa) were amplified from a human liver cDNA library (Takara) and cloned into a HisBioFLAG-pcDNA vector to form pcDNA-HisBioFlag-ASGR1ex or pcDNA-HisBioFlag-ASGR2ex. T7His-pcDNA vector was generated *via* In-Fusion PCR from HisBioFLAG-pcDNA using T7His-INV-F and T7His-INV-R primers. Ack1 cDNA was amplified from the total cDNA of HepG2 cells *via* PCR using Ack1-f and Ack1-r, and the PCR products were cloned into the plasmid vector T7His-pcDNA.

All amino acid sequences used in the construction of 10M-D42AN are listed in Table S2. 10M-D42AN was constructed by infusion cloning of the functional peptides.

### IVV library construction and screening for DOCK11-binding peptides

Construction of the 9- and 16-mer random DNA libraries was performed as previously described (51). Briefly, a random peptide library of 9 and 16 residues was amplified, transcribed, ligated with a PEG-puro spacer (52) with a 2-nitrobenzyl linker capable of photocleavage (42), and translated in a cell-free wheat germ translation system (ZoeGene). The 293T cells were transfected with DOCK11-DHR2-BioFLAGHis-pcDNA plasmid using Lipofectamine 2000 (Invitrogen) and then cultured for 24 h. The cells were washed with PBS and lysed with radioimmunoprecipitation assay (RIPA) buffer (Thermo) containing protease inhibitor cocktail (Nacalai Tesque). Lysates were centrifuged at 4 °C for 30 min at 13,200 rpm, and the supernatant was collected and applied to TALON magnetic beads (Clontech) at 4 °C overnight. DOCK11-DHR2-BioFLAGHis was eluted with Tris-buffered saline (TBS) containing 500 mM imidazole. Eluted proteins were immobilized on the Sensor Chip SA (Cytiva). The IVV library was subjected to the chip on a Biacore 3000 system (Cytiva), followed by irradiation with 365 nm UV light for 20 min at 4 °C (42). The recovered RNA was amplified, purified, and used for the next round of selection. After eight rounds of selection, the RT-PCR product was cloned and sequenced. HepG2-NTCP-C4-Halo-DOCK11 cells were infected with HBV particles at 20,000 GEq/cell on days 0 and 6. At day 15, the cells were induced with doxycycline to express DOCK11 overnight and then transfected with the plasmid to induce expression of the NLS-tagged DCS8-42A protein. After incubation for 72 h, the cells were collected to estimate infection.

### IVV library construction and screening for anti-ASGR antibody

The heavy chain and light chain of scFv were amplified from mouse spleen Poly A + RNA (Clontech) and ligated *via* overlap PCR (53, 54) to construct a mouse-derived single chain antibody cDNA library. The 293T cells were transfected with pcDNA-HisBioFlag-ASGR1ex or pcDNA-HisBioFlag-ASGR2ex plasmid using Lipofectamine 2000 and then cultured for 48 h. The cells were washed with PBS and lysed with cell lysis buffer (Tris–HCl pH 7.4 25 mM, NaCl 137 mM, KCl 2.68 mM, 1% Triton X-100). Lysates were applied to anti-FLAG M2 affinity gel (Sigma) at 4 °C overnight and eluted with 3× FLAG peptide (Sigma). Selection was performed as described previously. After three rounds of selection, the RT-PCR product was cloned and sequenced. Selected anti-ASGR antibodies were cloned into an IgK-pcDNA vector to construct IgK-ASGR3-10M. The 293T cells were transfected with the IgK-ASGR3-10M plasmid using Lipofectamine 2000 and then cultured for 6 days. The medium was applied to streptavidin magnesphere paramagnetic particles (Promega) immobilized with ASGR1ex or ASGR2ex on a rotator at 4 °C for 1.5 h. The beads were washed with TBS with Tween-20 (TBST) (TBS, pH 7.4 [Nacalai Tesque] with 0.1% Tween-20 [Nacalai Tesque]) and resuspended in NuPAGE LDS Sample Buffer (4×) (Invitrogen) containing 0.1 M DTT. The eluate was separated in 4 to 12% SDS-PAGE and analyzed by Western blotting with horseradish peroxidase (HRP)–conjugated mouse anti-FLAG-tagged monoclonal antibody (Sigma).

### Western blotting

Huh7 cells were treated with 100 nM 10M-D42AN for 0 to 48 h and with EGF (Invitrogen) (0–100 ng/ml) for 0 to 3 h. To observe the phosphorylation levels of Ack1 and WASP, HepG2 cells were transfected with T7-Ack1-His-pcDNA using Lipofectamine 2000 and then cultured for 48 h. These cells were lysed with RIPA buffer containing a protease inhibitor cocktail and phosphatase inhibitor cocktail. Lysates were centrifuged at 4 °C and 13,200 rpm for 15 min, and the supernatant was removed. Protein concentrations were determined using a bicinchoninic acid protein assay kit (Thermo). Equivalent amounts of protein were separated in NuPAGE 4% to 12% Bis–Tris gel (Thermo) or NuPAGE 3% to 8% Tris-acetate gel (Thermo) followed by incubation with primary antibodies against Ack1, EGFR, phosphorylated-EGFR Y845, pN-WASP (Santa Cruz), pAck1, GAPDH (Abcam, CST), Chk1, pChk1, phosphorylated-EGFR Y1045, Y1068, N-WASP (CST), and γH2AX (Merck) and with HRP-conjugated antibodies against FLAG, T7 (Novagen), and GST (Santa Cruz). HRP-conjugated secondary antibody against rabbit or mouse (Abcam, CST) was then applied. The blots were developed using enhanced chemiluminescence reagents (Nacalai Tesque, Cytiva).

### His-tag purification

HEK293T cells were transfected with DOCK11-BioFLAGHis-pcDNA or T7-Ack1-His-pcDNA using Lipofectamine 2000 or PEI Max (Polysciences) and then cultured for 48 to 72 h. Cells were washed with PBS and lysed with RIPA buffer containing protease inhibitor cocktail. Lysates were centrifuged at 4 °C for 30 min at 13,200 rpm, and the supernatant was collected and applied to a HisTrap HP column (Cytiva). The column was washed with buffer A (20 mM Tris–HCl, 500 mM NaCl, and 20 mM imidazole, pH 8.0), and then, His-tagged protein was eluted with buffer B (20 mM Tris–HCl, 500 mM NaCl, and 500 mM imidazole, pH 8.0) in gradient mode. The eluted fraction was concentrated using an Amicon Ultra-0.5 ml 10K Centrifugal Filter Unit (Millipore) followed by buffer exchange to TBS buffer (Nacalai Tesque).

### Furin digestion

scFv-peptide (a) 17 ng was mixed with 2 U of Furin protease (NEB) in cleavage buffer (200 mM Hepes (pH 7.5), 1% Triton X-100, 2 mM CaCl_2_, 2 mM 2-mercaptoethanol) at 37 °C for 30 min scFv-peptide (a) immobilized on His Mag Sepharose Excel (Cytiva) in PBS was mixed with human whole blood-K2EDTA at 37 °C for 0 to 6 h. These solutions were separated in a 4 to 12% SDS-PAGE and analyzed by Western blotting with HRP-conjugated mouse anti-FLAG-tagged monoclonal antibody or anti-T7-tagged antibody.

### Pull-down assay

DOCK11-BioFLAGHis purified as described previously was immobilized on streptavidin-conjugated magnetic beads (Promega) in TBST. Lysate containing T7-Ack1-His was mixed with DOCK11-immobilized beads on a rotator for 3 h at 4 °C in the presence of DCS8-42A. The beads were washed with TBST and resuspended in NuPAGE LDS Sample Buffer (4×) containing 0.1 M DTT. The eluate was separated in a 3 to 8% SDS-PAGE and analyzed by Western blotting with HRP-conjugated mouse anti-FLAG-tagged monoclonal antibody or anti-Ack1 antibody.

### Coimmunoprecipitation

HEK293T cells were transfected with DOCK11-BioFLAGHis or BioFLAGHis vector and T7-Ack1-His using Lipofectamine 2000 for 48 h. Cells treated with 10M-D42AN for 24 h were lysed with IP lysis buffer (Thermo) containing protease inhibitor cocktail and phosphatase inhibitor cocktail (Nacalai Tesque). Lysates were centrifuged at 4 °C for 15 min at 13,200 rpm, and the supernatant was applied to streptavidin-conjugated magnetic beads on a rotator for 3 h at 4 °C. The beads were washed with TBST and resuspended in NuPAGE LDS Sample Buffer (4×) containing 0.1 M DTT. The eluate was separated in a 3 to 8% SDS-PAGE and analyzed by Western blotting with HRP-conjugated mouse anti-FLAG-tagged monoclonal antibody or anti-Ack1 antibody.

### GST purification

*Escherichia coli* strain BL21 star (DE3) was transformed with the GST-Cdc42-pET plasmid. The cells were grown in LB at 37 °C. When *A*_600_ reached 0.4 to 0.5, 0.1 M IPTG was added, and the cells were cultured for another 2 h. The culture was centrifuged at 8500 rpm for 5 min. The pellet was lysed using GST buffer (20 mM Tris–HCl, pH 8.0, 1 mM EDTA, pH 8.0, 1% Triton X-100, 1 mM PMSF) containing 5 mM DTT and protease inhibitor cocktail and homogenized by sonication. The homogenate was centrifuged at 8500 rpm for 20 min at 4 °C. The supernatant was filtered through a 0.45 μm filter and collected as the soluble fraction.

The soluble fraction was added to glutathione sepharose 4B (Cytiva) and mixed by rotation at 4 °C overnight. The beads were washed with GST buffer containing 1 mM DTT, and the protein was then eluted with elution buffer (20 mM Tris–HCl, pH 8.0, 150 mM NaCl, 10 mM reduced glutathione, 0.1 mM EDTA, pH 8.0, 0.25 mM DTT, 0.1 mM PMSF, 25% glycerol). The resulting eluates were collected by centrifugation at 500*g* for 5 min at 4 °C.

### GEF assay

HEK293T cells cultured to 80% confluence were transfected with plasmid DOCK11-DHR2-BioFLAGHis-pcDNA using Lipofectamine 2000 and incubated for 48 h. The cells were rinsed with PBS followed by lysis with IP lysis buffer containing protease inhibitor cocktail, phosphatase inhibitor cocktail, and 0.1 M PMSF (CST). The lysates were applied to streptavidin-coated plates (Thermo) at 4 °C overnight. The 2× exchange buffer (40 mM MES-NaOH, pH 7.0, 300 mM NaCl, 20 mM MgCl2, 0.4 mg/ml bovine serum albumin, 40 μM GDP) and DCS8-42A prepared to 0 to 0.10 pmol were added at room temperature (RT) for 30 min in the dark. Purified GST-Cdc42 was mixed with 2× exchange buffer, pretreated at 30 °C for 8 min, and then added to the plate for 30 min in the dark. The plate was supplemented with 0.5 mM Mant-GTP to reach a final concentration of 2.5 μM, and the absorbance was immediately measured in a plate reader.

### Gene knockdown

Cells cultured to 80% confluence were transfected with Ack1 or DOCK11 siRNA #1-3 (Sigma) using Lipofectamine 3000 (Invitrogen) and incubated for 72 h for knockdown of the target protein.

### UV irradiation

HepG2 cells or PXB cells were transfected with siRNA targeting DOCK11 for 72 h or treated with 10M-D42AN for 24 h. The medium of these cells was removed, and the cells were irradiated with 140 J/m2 UV light using a handy UV lamp (Funakoshi) and then cultured in penicillin/streptomycin-free medium for an additional 1 h.

### Quantitative RT-PCR

Total RNA was extracted from cells using NucleoSpin RNA (Takara) and purified using Nucleospin RNA Clean-up XS (Takara). cDNA was synthesized by reverse transcription using a PrimeScript RT reagent Kit (Takara). Quantitative RT-PCR for *DOCK11* mRNA was performed using TB Green Premix Ex Taq II (Takara) and Thermal Cycler Dice Real Time System Lite TP700 (Takara). *GAPDH* mRNA was quantified and standardized in the same samples. Primer sets were as shown in Table S3.

### Microscopic observation

HepG2 cells or PXB cells on coverslips were treated with 10M-D42AN or GFP-tagged scFv-fused peptides. For observation of nuclear actin, the cells were transfected with plasmid EYFP-NLS-Actin (kindly provided by Prof. Harada, Tohoku University) using Lipofectamine 2000 and cultured for 72 h. For early endosome staining, CellLight Early Endosomes-RFP, BacMam 2.0 (Invitrogen) was used. These cells were then fixed with 10% paraformaldehyde for 10 min followed by permeabilization with 0.5% Triton X-100 (Nacalai Tesque) for 5 min. The samples were stained with antibodies against DOCK11 (kindly provided by Prof. Matsushima, Kanazawa University), pChk1, Ack1, and γH2AX followed by Alexa488-conjugated anti-rabbit IgG (Invitrogen) and Alexa647-conjugated antimouse IgG (Invitrogen). For staining of cytoplasmic actin filaments, 100 nM rhodamine phalloidin (Acti-Stain 535, Cytoskeleton) was used. The 4′,6-diamidino-2-phenylindole solution (Thermo) was used for nuclear staining. Photobleaching was prevented using Slow Fade Gold antifade reagent (Invitrogen). The samples were observed using confocal microscopy. The cells were observed with an LSM800 confocal microscope (Zeiss) and analyzed using ZEN software (Zeiss). The fluorescence intensity of the target protein was normalized to that of 4′,6-diamidino-2-phenylindole.

### Mice

All experiments performed in mice were approved by AAALAC International. SCID mice were constructed by PhoenixBio Co Ltd. Among the cDNA-uPAwild/+/SCID mice transfected with human hepatocytes, we used PXB mice with an expected replacement rate of human hepatocytes of more than 70%, calculated based on human albumin levels in mouse blood. Groups of PXB mice (5/group) were dosed with 10M-D42AN or PBS *via* i.p. administration at 300 μl/mouse once every 2 to 3 days from the eighth week of virus inoculation (day 0) to the day before specimen collection (day 27).

At necropsy (day 28), under isoflurane inhalation anesthesia, all available blood samples (>400 μl) were collected from the abdominal vena cava or heart. Two microliters of blood was diluted with 200 μl of saline and centrifuged at 390*g* and RT for 10 min. The concentration of h-Alb in blood was measured *via* latex agglutination immunoturbidimetry (LZ Test 'Eiken' U-ALB, Eiken Chemical) with an automated analyzer BioMajesty (JCA-BM6050, JEOL). Alanine aminotransferase was measured by Phoenix Biotechnology using 10 μl of serum from blood collected at autopsy. Diaryl imidazole leucopigment was measured using a DryChem 7000/NX500sV.

After euthanasia on day 28, the livers were collected and weighed. DNA was extracted from liver samples immersed in RNAlater using DNeasy Blood & Tissue Kits (Qiagen), and DNA was dissolved in nuclease-free water to a final concentration of 20 ng/μl. PCR solution was prepared using 5 μl of dissolved DNA stock solution or diluted DNA and TaqMan Fast Advanced Master Mix (Thermo). PCR for HBV DNA or cccDNA was performed with 53 to 55 cycles of 95 °C for 3 s and 60 °C for 32 s. The sequences of the primers and probes used are shown in Table S4. Serum obtained from HBV-infected PXB mice was used as the HBV DNA standard.

## Data availability

All correspondence and material requests should be addressed to corresponding author, H. Y.

## Supporting information

This article contains supporting information.

## Conflict of interest

The authors declare that they have no conflicts of interest with the contents of this article.
