# Supplementary material for: Guanine nucleotide exchange factor DOCK11-binding peptide fused with a single chain antibody inhibits hepatitis B virus infection and replication
Source: J Biol Chem. 2022 Jun 2;298(7):102097. doi: 10.1016/j.jbc.2022.102097 (PMC9241042; doi:10.1016/j.jbc.2022.102097)
Supplement: Supplemental Figure S1 [file mmc2.pdf]

Figure S1.

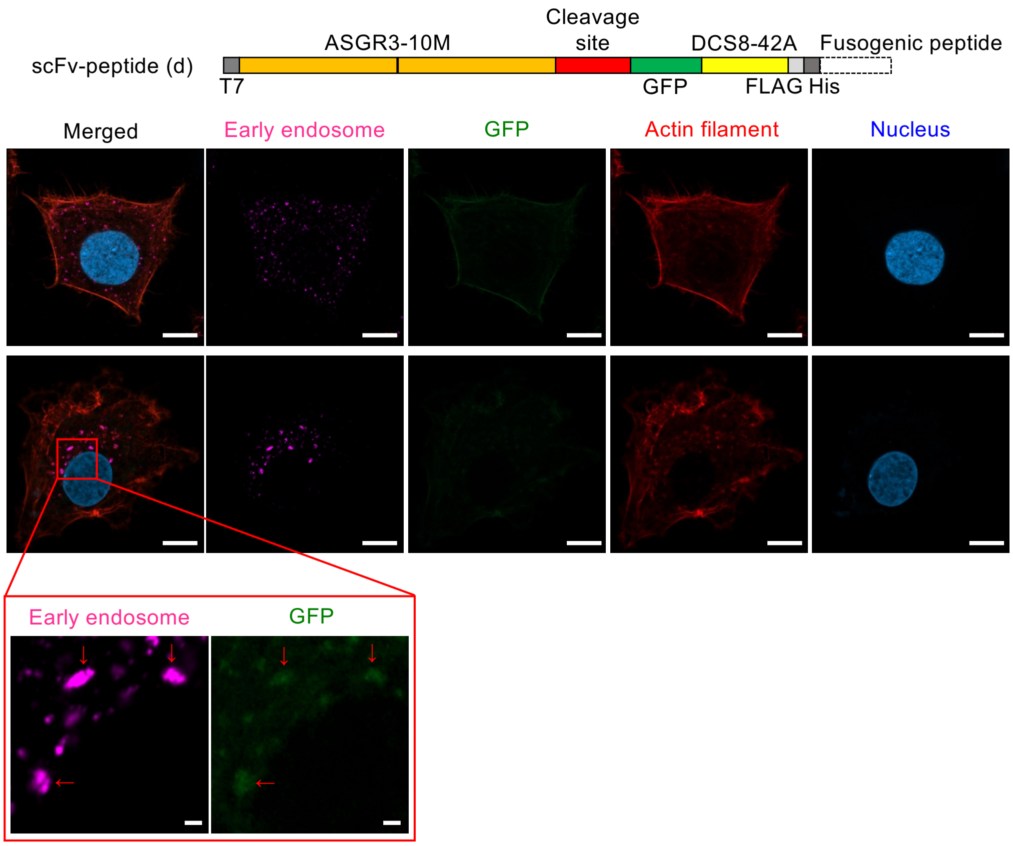

Figure S1.

HepG2 cells were treated with scFv-peptide (d) that does not contain the fusogenic peptide. After 2-5 h, immunofluorescence staining with CellLight Early Endosomes-RFP (pink) and rhodamine phalloidin (red) was performed. Scale bars, 10 μm. An enlarged image of the area enclosed by the red square in the figure is shown on the right (Scale bars, 1 μm).
