# Supplementary material for: Guanine nucleotide exchange factor DOCK11-binding peptide fused with a single chain antibody inhibits hepatitis B virus infection and replication
Source: J Biol Chem. 2022 Jun 2;298(7):102097. doi: 10.1016/j.jbc.2022.102097 (PMC9241042; doi:10.1016/j.jbc.2022.102097)
Supplement: Supplemental Figure S2 [file mmc3.pdf]

Figure S2.

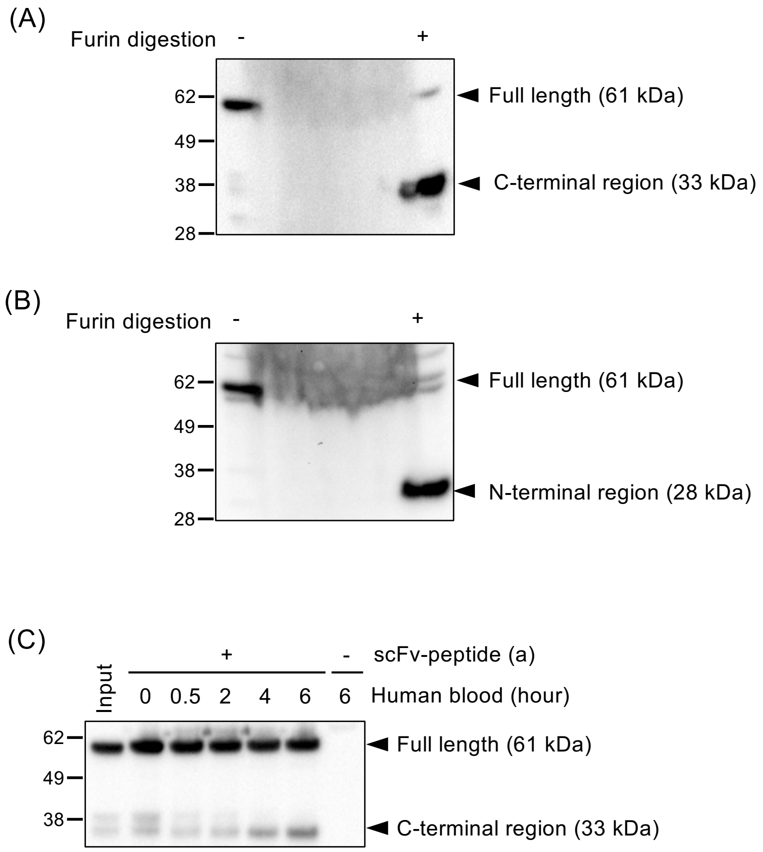

Figure S2.

(A and B) scFv-peptide (a) was treated with the protease Furin at 37 ° C for 30 min, separated in a 4-12% SDS–PAGE and analyzed by western blotting with antibodies against FLAG-tag (A) and T7-tag (B).

(C) scFv-peptide (a) was added to human blood at 37 ° C for 0-6 hours, separated in a 4-12% SDS–PAGE and analyzed by western blotting with an antibody against FLAG-tag.
