# Supplementary material for: Guanine nucleotide exchange factor DOCK11-binding peptide fused with a single chain antibody inhibits hepatitis B virus infection and replication
Source: J Biol Chem. 2022 Jun 2;298(7):102097. doi: 10.1016/j.jbc.2022.102097 (PMC9241042; doi:10.1016/j.jbc.2022.102097)
Supplement: Supplemental Figure S3 [file mmc4.pdf]

Figure S3.

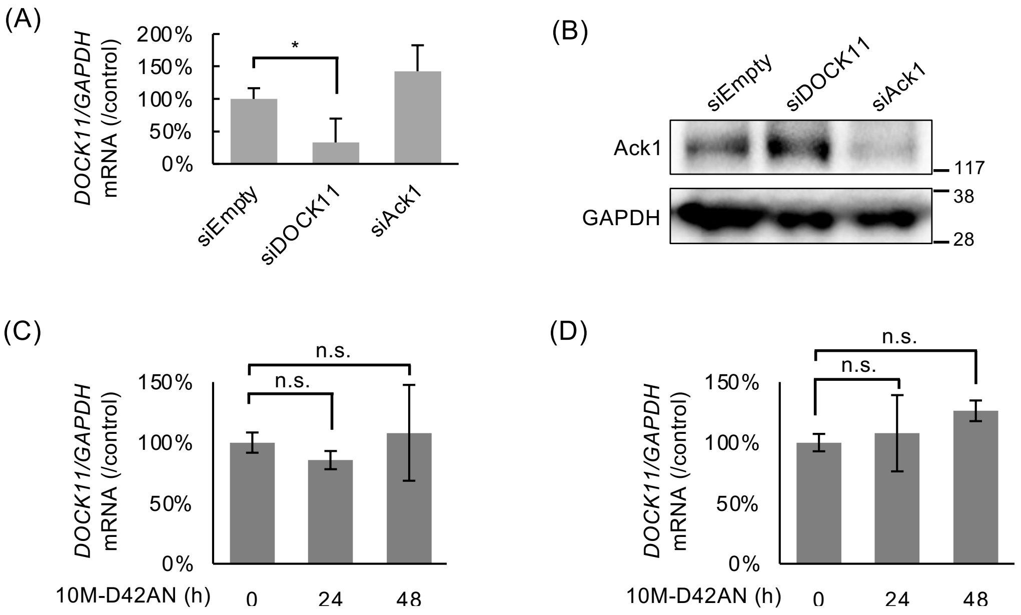

Figure S3.

(A) The amount of DOCK11 mRNA in HepG2 cells treated with siRNA targeting DOCK11 or Ack1. Total RNA was extracted and then amplified via RT–PCR with specific primers for GAPDH or DOCK11. Data are presented as the mean ± SD pooled from three independent experiments. *$p < 0.05$.

(B) The endogenous Ack1 expression level in HepG2 cells treated with siRNA targeting DOCK11 or Ack1 were analyzed by western blotting with an antibody against Ack1. GAPDH is shown to verify equal loading.

(C, D) The amount of DOCK11 mRNA in HepG2 cells (C) or Huh7 cells (D) treated with 10M-D42AN for 0-48 h. Total RNA was extracted and then amplified via RT–PCR with specific primers for GAPDH or DOCK11. Data are presented as the mean ± SD pooled from three independent experiments. n.s.: not significant.
