# Supplementary material for: Guanine nucleotide exchange factor DOCK11-binding peptide fused with a single chain antibody inhibits hepatitis B virus infection and replication
Source: J Biol Chem. 2022 Jun 2;298(7):102097. doi: 10.1016/j.jbc.2022.102097 (PMC9241042; doi:10.1016/j.jbc.2022.102097)
Supplement: Supplemental Figure S4 [file mmc5.pdf]

Figure S4.

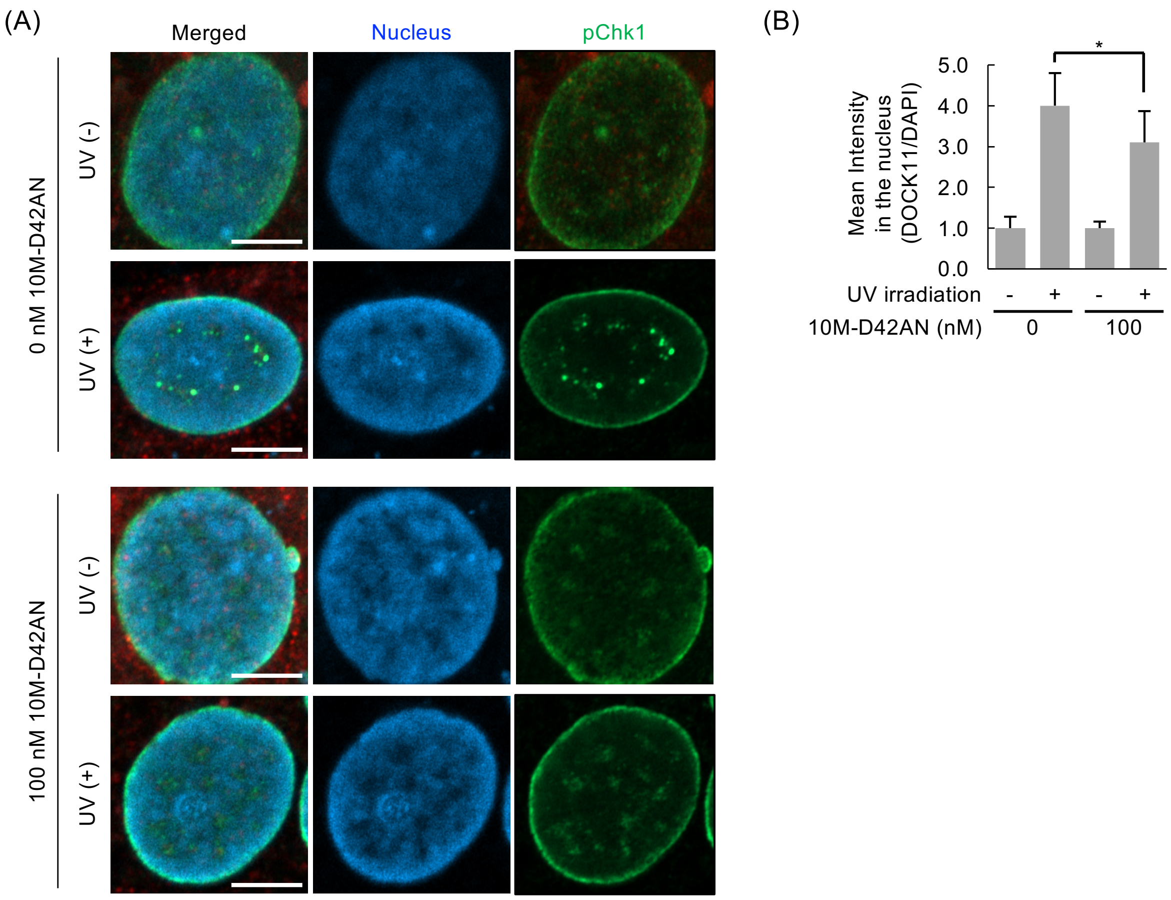

Figure S4.

(A) Immunofluorescence staining of PXB cells using anti-DOCK11 antibody (Alexa488, green) after treatment with 0-100 nM 10M-D42AN for 24 h and UV irradiation. The samples were observed under a fluorescence microscope. Scale bars, 10 μm.

(B) The fluorescence intensity of DOCK11 in the nucleus was quantified and normalized to that of DAPI. The ratio under the indicated conditions is shown. Data are presented as the mean ± SD pooled from three independent experiments. *$p < 0.05$.
