# Supplementary material for: Guanine nucleotide exchange factor DOCK11-binding peptide fused with a single chain antibody inhibits hepatitis B virus infection and replication
Source: J Biol Chem. 2022 Jun 2;298(7):102097. doi: 10.1016/j.jbc.2022.102097 (PMC9241042; doi:10.1016/j.jbc.2022.102097)
Supplement: Supplemental Figure S5 [file mmc6.pdf]

Figure S5.

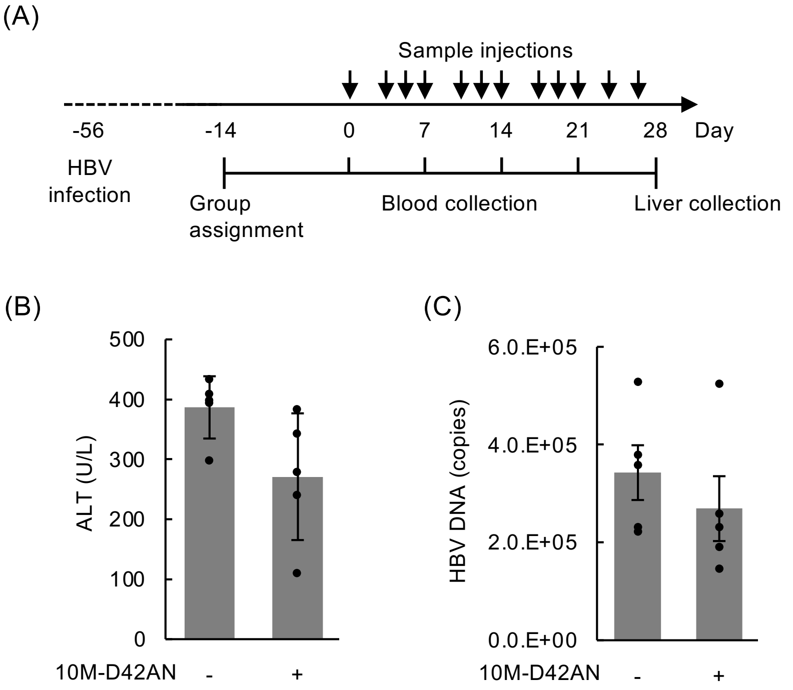

Figure S5.

(A) Schedule of HBV infection and 10M-D42AN injection in PXB mice (PhoenixBio). (B) ALT in the serum at autopsy was measured with diaryl imidazole leucopigment. (C, D) HBV DNA (C) and cccDNA (D) in the liver were detected by PCR.
